# Exploring Alexithymia, Uncertainty, Anxious Arousal, and Social Anxiety as Mediators of the Relationship Between Sensory Processing Differences and Restricted and Repetitive Behaviors in Autistic Adults

**DOI:** 10.1002/aur.70145

**Published:** 2025-11-21

**Authors:** Heather L. Moore, Samuel Brice, Natalya Spraggon, Barry Ingham, Mark Freeston, Jeremy R. Parr, Jacqui Rodgers

**Affiliations:** ^1^ School of Psychology Newcastle upon Tyne UK; ^2^ Cumbria, Northumberland, Tyne & Wear NHS Foundation Trust, St Nicholas Hospital Newcastle upon Tyne UK; ^3^ Population Health Sciences Institute, Newcastle University, Sir James Spence Institute, Royal Victoria Infirmary Newcastle upon Tyne UK

**Keywords:** alexithymia, anxious arousal, autism, restricted and repetitive behaviors, sensory processing, social anxiety, uncertainty

## Abstract

Restricted and repetitive behaviors (RRB) are associated with sensory processing (SP) differences for autistic people, and are thought to be a coping strategy to help manage the sensory environment. Previous work shows that, for autistic people, alexithymia, intolerance of uncertainty (IU), and anxiety mediate the relationship between SP differences and RRB. However, these studies use anxiety measures developed for the general population, and more recent evidence suggests that autistic people may have a different anxiety experience. This study aims to extend previous findings by unpacking the anxiety experience for autistic adults in the relationship between SP differences and RRB, using an autism‐specific anxiety measure. Data were available from 426 autistic adults. Serial mediation models tested the relationship between SP differences and RRB, with alexithymia, IU, anxious arousal, and social anxiety as mediators. We identified significant direct effects from SP differences to both repetitive motor behaviors (RMB) and insistence on sameness behaviors (ISB). For RMB, we found indirect effects through anxious arousal, alexithymia‐anxious arousal, IU‐anxious arousal, and alexithymia‐IU‐anxious arousal. For ISB, we found indirect effects through IU and alexithymia‐IU. Thus, different mechanisms may underpin RMB and ISB. Understanding the anxiety experience of autistic people, alongside the role of SP and RRB, is key to providing tailored support, adjustments, and psychological interventions to autistic people. Future research could benefit from directly investigating the impact of strategies to support SP and anxiety.

## Summary

Restricted and repetitive behaviors (RRB) are linked to sensory processing and are thought to be a coping strategy to help manage the sensory environment. Difficulties with emotions are associated with anxiety from the sensory environment. Repetitive motor behaviors (RMB) may help manage anxious arousal brought on by the sensory environment. Insistence on sameness behaviors (ISB) may help cope with intolerance of uncertainty brought on by the sensory environment. We should consider the sensory experience, anxiety experience, and coping strategies for these when offering support to autistic people.

## 1 Introduction

Autistic individuals often experience sensory under‐ and/or over‐responsivity throughout the lifecourse (Crane et al. 2009; Germani et al. 2014; Kern et al. 2006). Restricted and repetitive behaviors (RRB) are suggested as a coping mechanism to manage sensory processing (SP) differences (Gal et al. 2002; Glod et al. 2019; Moore et al. 2021; Schulz and Stevenson 2019, 2020). RRB comprise repetitive motor behaviors (RMB; including repetitive language, movements, spinning, tapping) and insistence on sameness behaviors (ISB; including invariance from routines and circumscribed interests). RMB may represent attempts to increase sensory stimulation, or block other unwanted sensory input, for example, using repetitive language and movements (Baker et al. 2008; Kapp et al. 2019); whereas ISB may represent attempts to impose predictability and consistency, and reduce uncertainty in relation to exposure to aversive sensory input, through the use of routines and familiar interests (Lidstone et al. 2014).

Mental health conditions, such as anxiety, are commonly experienced by autistic people (Hollocks et al. 2018; Hossain et al. 2020; Lai et al. 2019). Intolerance of uncertainty (IU) is a transdiagnostic mechanism associated with anxiety (Carleton et al. 2012, 2007; Jenkinson et al. 2020), defined as the tendency to experience distress about unknown experiences, regardless of whether possible outcomes are negative (Freeston et al. 2020). Previous research has demonstrated a mediating role of IU and anxiety in the relationship between SP and RRB, alongside alexithymia (Moore et al. 2021). Both IU and anxiety are associated with SP and RRB, individually and in conjunction with each other (Glod et al. 2019; Hwang et al. 2020; Wigham et al. 2015). Alexithymia, another condition commonly experienced by autistic people (Kinnaird et al. 2019), is characterized by difficulties identifying and describing emotions (Nemiah 1996). It is associated with SP differences, IU, and anxiety (Liss et al. 2008; Maisel et al. 2016; Pickard et al. 2020). In the largest study to date investigating these relationships (*N* = 426 autistic adults), Moore et al. (2021) found significant direct effects of SP differences on both RMB and ISB. For SP to RMB, there were serial mediating effects through alexithymia‐IU‐anxiety. For SP to ISB, only IU as a single mediator and alexithymia‐IU as a serial mediator were significant; alexithymia was not involved. The differences in findings between ISB and RMB demonstrated that the role of alexithymia was through its relationship with IU, and anxiety was not a significant mediator for ISB, in contrast to previous child literature (Black et al. 2017; Factor et al. 2016; Rodgers et al. 2012; Uljarević et al. 2017).

While Moore et al.'s work demonstrated the differential associations of alexithymia, IU, and anxiety in the relationship between SP and RRB, their conclusions are limited by the use of an anxiety measure developed for the general population: the Hospital Anxiety and Depression Scale (HADS; Zigmond and Snaith 1983). There is increasing evidence that some autistic people may have a different anxiety experience from non‐autistic people (Brice, Welsh, et al. 2021; Kerns and Kendall 2012; Ollendick and White 2012; Renno and Wood 2013). Emerging theoretical frameworks encompass the empirical findings of autism‐specific anxiety correlates, identifying SP, alexithymia, and IU as predictors of anxiety (Riedelbauch et al. 2023; South and Rodgers 2017; Stark et al. 2021), alongside other factors such as worries about routine, novelty, and restricted interests; unusual phobias; social anxiety related to negative self‐evaluation more than fear of rejection; and compulsive behavior not motivated by attempts to relieve distress (Kerns et al. 2014; Rodgers et al. 2023). Psychometric evidence supports this variation. Systematic investigation of a range of anxiety measures developed for the general population postulates that some tools are adequate for autistic individuals (including the HADS used by Moore et al. (2021)), but may provide fluctuating anxiety prevalence rates (Hollocks et al. 2018; Trembath et al. 2012), and autism‐specific measures would be more appropriate (Kerns et al. 2015; Lecavalier et al. 2014; Uljarević et al. 2018; Wigham and McConachie 2014). Thus, measures that only include items associated with neurotypical experiences of anxiety may be potentially suboptimal for capturing the experiences of autistic people.

A recently developed anxiety measure for autistic adults captures three facets: Uncertainty, Anxious Arousal, and Social Anxiety (Rodgers et al. 2020). Uncertainty represents IU definitions described previously. Anxious arousal, also known as somatic anxiety, is a physiological anxiety experience, characterized by symptoms, such as dizziness, shortness of breath, increased heart rate, and nervous stomach, alongside heightened reactivity toward physiological symptoms and perceived environmental threat monitoring (Nitschke et al. 1999). Social anxiety disorder is defined by the DSM‐5 as an intense fear of social or performance situations in which a person may be exposed to unfamiliar people or possible scrutiny by others, leading to anxiety and/or avoidance that interferes with daily life (American Psychiatric Association 2013). Given previous associations identified between SP, RRB, and anxiety in autistic adults (Moore et al. 2021), it would be useful to explore how facets of anxiety associated with autism impact the relationship between SP and RRB.

Of these three facets of anxiety, IU from the Uncertainty factor has been most comprehensively investigated with autistic individuals, and clear relationships have already been described between uncertainty, SP, and RRB (e.g., Glod et al. 2019; Hwang et al. 2020; Maisel et al. 2016; Moore et al. 2021; Wigham et al. 2015). Little work has explored the relationship between anxious arousal and social anxiety in SP and RRB. Limited evidence shows heightened physiological arousal in response to SP differences (Krusemark and Li 2012; Miller et al. 1999). While regulating arousal has long been proposed as a potential function of RRB, to the author's knowledge, only one study with children has empirically tested this theory. Joosten et al. (2009) found that teacher‐reported arousal was a motivator for RRB in autistic children with intellectual disabilities. Conflicting evidence exists regarding the relationship between social anxiety and both SP and RRB. Black et al. (2017) found no relationship between parent‐reported SP and social anxiety (*N* = 79 autistic children), whereas Pickard et al. (2020) showed significant, strong, positive correlations between hypersensitivity and social anxiety in both autistic (*N* = 61) and non‐autistic (*N* = 62) adolescents, using self‐report. Hyposensitivity was more weakly associated in the autistic group, and nonsignificant in the non‐autistic group. In contrast, MacLennan et al. (2020) demonstrated a negative relationship between hyposensitivity and social anxiety in 3‐ to 14‐year‐old children (*N* = 41). Finally, Riedelbauch et al. (2023) found significant positive correlations between SP and social anxiety in autistic (*N* = 86) and non‐autistic (*N* = 100) adults. Considering the relationship between social anxiety and RRB, Schiltz and Magnus (2021) found increased social anxiety significantly related to higher RRB in 198 autistic 5‐ to 17‐year‐olds, whereas Ben‐Itzchak et al. (2020) found the reverse, with increased social anxiety associated with decreased RRB in a smaller sample of autistic youth (*N* = 61, 10–18 years). Overall, these findings demonstrate evidence, though variable, for the relationship of uncertainty, anxious arousal, and social anxiety with SP and RRB in autistic individuals.

It is important to also consider how these three facets of anxiety relate to each other, and alexithymia, in the relationship between SP and RRB. As described earlier, IU is associated with alexithymia and anxiety in the relationship between SP and RRB (Glod et al. 2019; Hwang et al. 2020; Maisel et al. 2016; Moore et al. 2021; Wigham et al. 2015). But how do anxious arousal and social anxiety fit in?

First, considering anxious arousal, studies have demonstrated increased aversive physiological reactions in response to uncertainty, in both the general population and IU samples (e.g., Chen and Lovibond 2016; Chin et al. 2016; Grupe and Nitschke 2011). Using self‐report in non‐autistic children, IU at time‐1 predicted self‐reported anxious arousal at time‐2 (Panarello and Bukowski 2021). Top et al. (2019) found evidence for chronic hyper‐arousal in autistic compared to non‐autistic adults, alongside stronger correlations between SP differences and IU. Moreover, qualitative research with parents describes arousal reduction to manage uncertainty in their autistic children (Hodgson et al. 2017). While not measuring anxious arousal specifically, Larkin et al. (2022) found that alexithymia and IU predict general somatic symptoms (e.g., pain, discomfort, dizziness, headache, and gastrointestinal symptoms) in autistic (diagnosed *N* = 51, self‐identifying *N* = 32) and non‐autistic adults (*N* = 119). Alexithymia also mediated the relationship between autistic traits and anxious arousal in a general population sample of 292 adults (Barros et al. 2022). This suggests a possible pathway from alexithymia to IU to anxious arousal, although it has yet to be verified among autistic people.

Evidence from non‐autistic populations has also demonstrated the relationship between IU and social anxiety (e.g., Boelen and Reijntjes 2009; Carleton et al. 2010; Whiting et al. 2014), and alexithymia and social anxiety (e.g., Edel et al. 2010; Evren and Evren 2007; Radetzki et al. 2021), but little research has explored this in autistic populations. Although underpowered for the analyses (*N* = 123), Pickard et al. (2020) found that child‐reported IU and sensory hypersensitivity mediated the relationship between autistic traits and social anxiety in a combined sample of autistic and non‐autistic adolescents, but alexithymia did not. Furthermore, physiological arousal (alongside social skills) predicted social anxiety in 41 autistic adolescents (Bellini 2006). Shapiro et al. (2020) found that IU predicted physiological arousal as well as the behavioral avoidance component of social anxiety individually, using a large outpatient psychiatric sample, but serial relationships were not considered. Finally, interventions for reappraisals of anxious arousal have demonstrated reductions in socially related anxiety among non‐autistic adults (Beltzer et al. 2014). While there may be feedback loops at play with IU and anxious arousal in the maintenance of social anxiety (Siess et al. 2014), this evidence suggests pathways through IU and anxious arousal to social anxiety.

To date, no research has simultaneously explored the relationships between SP, RRB, alexithymia, IU, anxious arousal, and social anxiety. This study aimed to extend the findings of Moore et al. (2021) to further unpack the role of anxiety (alongside alexithymia) in the relationship between SP and ISB/RMB for autistic adults; namely uncertainty, anxious arousal, and social anxiety. We predict that there will be a:

– Significant direct effect of SP differences on ISB and RMB, respectively.
– Significant indirect effect (mediation) by alexithymia, IU, anxious arousal, and social anxiety. We will explore individual and/or serial mediators.

**FIGURE 1:**
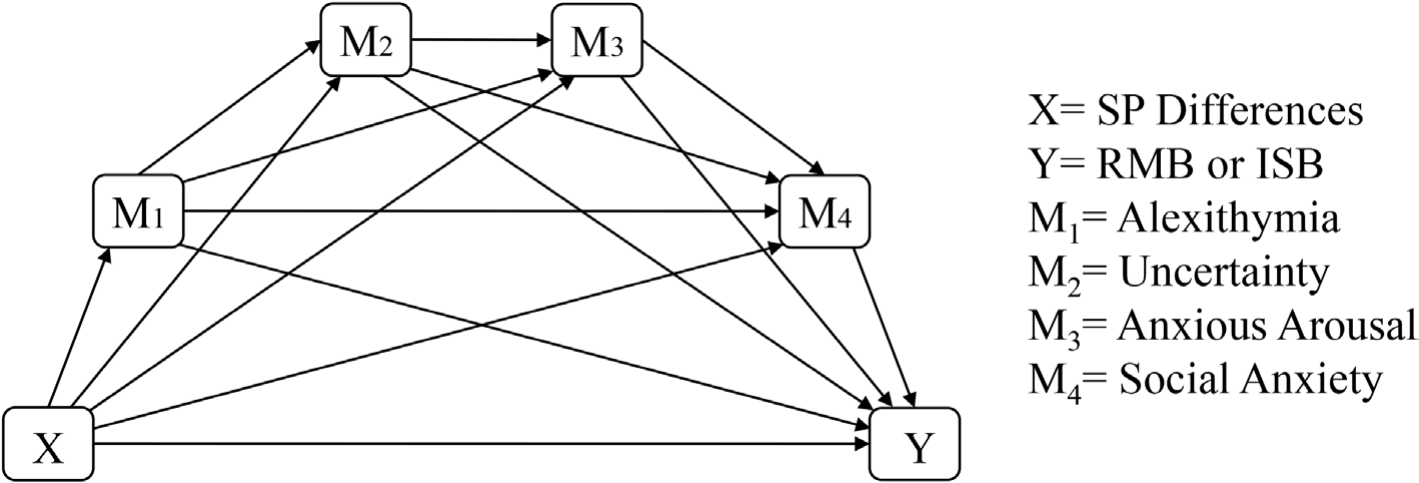
Conceptual diagram of Model 6 (four serial mediators) in Hayes PROCESS, indicating the direct and indirect pathways tested in this study. X = sensory processing differences; Y = repetitive motor behaviors, or insistence on sameness behaviors; M1 = alexithymia; M2 = uncertainty; M3 = anxious arousal; and M4 = social anxiety.

Figure 1 indicates the pathways tested in this study.

## 2 Methods

### 2.1 Participants

Data were obtained from 426 participants recruited to the Personalized Anxiety Treatment in Autism (PAT‐A) study, via the Adult Autism Spectrum Cohort‐UK (ASC‐UK; https://research.ncl.ac.uk/adultautismspectrum/). Depending on pre‐specified contact preferences, eligible participants from ASC‐UK were sent study information via email or letter (including a more accessible, short version). Inclusion criteria were autistic adults who were part of the ASC‐UK cohort, aged 18+ years, living in the United Kingdom, who self‐reported a diagnosed or suspected anxiety condition when they joined the cohort. Cohort participants included those who suspected they were autistic or were awaiting assessment (Brice, Rodgers, et al. 2021; Moore et al. 2021). This study used the same dataset as Moore et al. (2021). Table 1 shows participant demographic characteristics. There were no significant differences in key demographic variables, such as age, gender, or autism characteristics, between responders and non‐responders to the PAT‐A study invitation (see Brice, Rodgers, et al. 2021). Of the 426 respondents, 343 completed the survey online, and 83 completed and returned a paper copy of the survey.

**TABLE 1:** Demographic characteristics of the sample (*N* = 426).

Variable	
Age: Mean (SD) (range)	42.62 (13.89) (16–77)
Gender, *N* (%)	
Male	192 (45.07)
Female	223 (52.35)
Self‐describing	11 (2.58)
ASD diagnosis, *N* (%)	
Diagnosis	368 (86.38)
Suspected/awaiting	58 (13.62)
Ethnicity (*N* = 413), *N* (%)	
White	388 (93.95)
Mixed race	9 (2.18)
Asian	2 (0.48)
Black	1 (0.24)
Rather not say	9 (2.18)
Other	4 (0.97)
Employment status^a^ (*N* = 423), *N* (%)	
Employed no support	156 (36.88)
Employed with support	9 (2.13)
Retired	20 (4.73)
Unemployed	183 (43.26)
Volunteer	36 (8.51)
Other	19 (4.49)
Highest qualification, *N* (%)	
No formal	24 (5.63)
Basic skills	19 (4.46)
GCSE	81 (19.01)
A level	66 (15.49)
Certificate of higher education	15 (3.52)
Diploma of higher education	32 (7.51)
Bachelor's degree	109 (25.59)
Postgraduate degree	75 (17.61)
Other	5 (1.17)
SRS‐2 score (*N* = 379): Mean (SD) (range)	112.13 (25.83) (17–195)

^a^ Multiple responses possible; percentages reported as a proportion of the total *N*.

The PAT‐A study received a favorable ethical opinion from the NHS HRA and Wales REC 5 (ref: 18/WA/0014). All participants provided paper‐based or online written informed consent.

### 2.2 Measures

#### 2.2.1 Autistic Characteristics: Social Responsiveness Scale‐2 (SRS‐2)

The SRS‐2 is a 65‐item, self‐report measure of social functioning to characterize features associated with autism (Constantino and Gruber 2012). Items are rated on a 4‐point Likert scale from 1 (*not true*) to 4 (*almost always true*), then recoded to 0–3, such that scores range from 0 to 195, with higher scores indicating more severe symptoms. The SRS‐2 has excellent internal consistency (*α* = 0.94–0.96) in autistic and non‐autistic samples (Bruni 2014), with good predictive validity (sensitivity = 0.86 and specificity = 0.60; Mandell et al. 2012). In our sample, SRS‐2 internal consistency was good (*α* = 0.85).

#### 2.2.2 SP Differences: Sensory Preferences Questionnaire (SPQ)

The SPQ (Kent 2014) was adapted from the Diagnostic Interview for Social and Communication Disorders (DISCO; Wing et al. 2002) into a 21‐item, self‐report questionnaire to measure SP differences in adults. The SPQ is rated on a 5‐point Likert scale from 1 (*almost never*) to 5 (*almost always*), with total scores ranging from 21 to 105. Higher scores indicate more SP differences, with no reported cutoffs for impairment. Kent (2014) established good internal consistency in autistic samples (*α* = 0.89), as well as external validity, demonstrated by large correlations with the Adult/Adolescent Sensory Profile (Brown and Dunn 2002) sensory seeking (*r* = 0.69), sensory sensitivity (*r* = 0.75), and sensory avoidance (*r* = 0.74) quadrants, but not the low registration quadrant (*r* = −0.23). SPQ internal consistency was good (*α* = 0.87) in this sample.

#### 2.2.3 RRB: Adult Repetitive Behavior Questionnaire‐2 (RBQ‐2A)

The RBQ‐2A is a 20‐item adult self‐report measure of RRB (Barrett et al. 2015). Response options vary, but items are scored on a Likert scale from 1 to 3/4 (4 is collapsed into 3). A two‐factor structure representing RMB and ISB is supported in autistic samples (Barrett et al. 2018), and this solution shows acceptable–good internal consistency (RMB *α* = 0.70, ISB *α* = 0.81). Internal consistency for our sample was acceptable for RMB (*α* = 0.74) and ISB (*α* = 0.78). RMB total scores range from 7 to 21, ISB from 11 to 33. While there are no published cutoffs for impairment, higher scores indicate increased RRB presentation.

#### 2.2.4 Alexithymia: Toronto Alexithymia Scale (TAS‐20)

The TAS‐20 is a 20‐item self‐report questionnaire investigating the presence of alexithymia (Bagby et al. 1994). The items are rated on a 5‐point Likert scale from 1 (*strongly disagree*) to 5 (*strongly agree*), and total scores range from 20 to 100. Scores ≤ 51 indicate that alexithymia is not present, 52–60 indicate possible alexithymia, and ≥ 61 indicate alexithymia. With undergraduates, the measure demonstrated good internal consistency (*α* = 0.81) and test–retest reliability (*r* = 0.77; Bagby et al. 1994). The TAS‐20 has also demonstrated good convergent validity and test–retest reliability with autistic participants (Berthoz and Hill 2005). In this sample, TAS‐20 internal consistency was good (*α* = 0.83).

#### 2.2.5 Anxiety: Anxiety Scale for Autism‐Adults (ASA‐A)

The ASA‐A is a 20‐item self‐report measure designed to capture the autism‐relevant anxiety facets of Uncertainty (5 items), Anxious Arousal (9 items), and Social Anxiety (6 items) (Rodgers et al. 2020). Items are scored on a 4‐point Likert scale, from 0 (*never*) to 3 (*always*). Total scores range from 0 to 60 (Uncertainty: 0–15; Anxious Arousal: 0–27; and Social Anxiety: 0–18), and scores of ≥ 28 indicate the presence of significant levels of anxiety. In an autistic population, internal consistency was excellent for each factor (Uncertainty *α* = 0.83; Anxious Arousal *α* = 0.85; and Social Anxiety *α* = 0.85; Rodgers et al. 2020). Furthermore, test–retest reliability was excellent (0.82), and convergent and divergent validity were robust (HADS‐Anxiety *r* = 0.70; HADS‐Depression *r* = 0.47; Rodgers et al. 2020). In this sample, internal consistency was good for all factors (Uncertainty *α* = 0.83; Anxious Arousal *α* = 0.85; and Social Anxiety *α* = 0.85).

### 2.3 Procedure

Those who wished to participate completed questionnaires relating to their anxiety experiences and related constructs. Following demographic information, participants completed the questionnaires in the following order: SRS‐2; ASA‐A; TAS‐20; RBQ‐2A; and SPQ, alongside other measures not reported here. Participants were offered support to complete the questionnaires, and could skip items/questionnaires, take breaks, and return to the survey later. Participants contacted by letter did so using a prepaid return envelope, and those contacted by email completed the consent form and measures using Qualtrics (Qualtrics 2005).

### 2.4 The Involvement of Autistic People in This Research

Research priorities of the autism community are to improve understanding and treatment of anxiety experienced by autistic people (Cusack and Sterry 2016). Patient and public involvement groups of autistic people and other community stakeholders discussed and agreed on the methods and materials for the PAT‐A study, and an autistic person and a relative of an autistic person were co‐investigators.

### 2.5 Statistical Analysis

Data were prepared and analyzed using IBM SPSS Statistics Version 28 (IBM Corp 2021). SRS‐2, RBQ‐2A, and TAS‐20 items were reverse scored and/or recoded, according to their respective manuals (Bagby et al. 1994; Barrett et al. 2015; Constantino and Gruber 2012) and total scores were calculated for autistic traits (SRS‐2), SP differences (SPQ), alexithymia (TAS‐20), and anxiety (ASA‐A), alongside subscale scores for ASA‐A (Uncertainty, Anxious Arousal, and Social Anxiety) and RBQ‐2A (RMB and ISB). Where < 20% of items were missing for a variable for a participant (Peng et al. 2006), person‐mean substitution was used to calculate a total score based on that individual's mean score (Peyre et al. 2011). Missing values analysis revealed that 0%–6.81% of the data were missing for the variables selected for mediation analysis, and Little's MCAR test was nonsignificant (*χ* ^2^(108) = 122.48, *p* = 0.161), suggesting the data are likely to be missing completely at random. No outliers were identified. There was no evidence of substantial skew or kurtosis.

To confirm the appropriateness of using a combined sample, we compared SRS‐2 total scores of individuals with a formal (*M*: 111.82, SD: 25.92) vs. suspected (*M*: 113.98, SD: 25.48) diagnosis, as has been supported in previous research using the PAT‐A sample (Brice, Rodgers, et al. 2021; Moore et al. 2021). No differences were found between groups (*t*[377] = −0.57, *p* = 0.570), supporting the use of a combined sample.

Prior to mediation analyses, we conducted Pearson, point‐biserial, and Spearman correlations between variables, and demographic characteristics (age, gender (dummy coded into two variables: Woman, Man; reference variable: Self‐Describing), and highest qualification attained). We ran serial mediation analysis using Model 6 in PROCESS v4.2 (Hayes 2013) macro for SPSS, to test the direct effects (SP differences ➔ RMB/ISB), as well as individual and serial indirect pathways (Alexithymia, Uncertainty, Anxious Arousal, and Social Anxiety). Figure 1 describes the order of entry of each variable into the model. Age and gender were entered as covariates. We used percentile bootstrapping with 10,000 resamples at 95% upper and lower confidence intervals. Nonsignificant pathways were indicated by confidence intervals overlapping 0; standardized *B* values represent effect sizes.

## 3 Results

Table 2 shows descriptive statistics for each variable. 12.2% showed no signs of alexithymia, 21.1% showed possible alexithymia, and 66.7% met cutoffs for the presence of alexithymia. Anxiety total scores identified the presence of significant levels of anxiety in 79.1% of the sample. Other scales do not provide cutoffs, but the mean score as a percentage of the maximum score was 49.8% for SP Differences, 61.2% for RMB, 73.3% for ISB, 70.8% for Uncertainty, 41.4% for Anxious Arousal, and 71.2% for Social Anxiety.

**TABLE 2:** Descriptive statistics for measures of sensory processing (SP) differences, repetitive motor behaviors (RMB), insistence on sameness behaviors (ISB), alexithymia, ASA‐A total anxiety score, Uncertainty, Anxious Arousal, and Social Anxiety.

	*N*	Mean	SD	Min	Max
SP Differences	419	52.28	14.04	2	97
RMB	420	12.85	3.14	7	21
ISB	420	24.20	4.42	12	33
Alexithymia	418	64.76	11.99	20	92
ASA‐A Total	422	34.58	9.95	0	60
Uncertainty	422	10.62	3.14	0	15
Anxious Arousal	419	11.19	5.11	0	27
Social Anxiety	421	12.81	3.99	0	18

Abbreviation: ASA‐A, anxiety scale for autism‐adult.

Table 3 shows correlations between variables and demographic characteristics. SP Differences, RMB, and ISB showed large, positive correlations with each other, and medium, positive correlations with Uncertainty and Anxious Arousal. SP differences and RMB showed medium, positive correlations with Alexithymia and small, positive correlations with Social Anxiety, whereas ISB showed a small, positive correlation with Alexithymia and a medium, positive correlation with Social Anxiety. Alexithymia showed medium, positive correlations with all anxiety variables, and the anxiety variables showed medium, positive correlations with each other.

**TABLE 3:** Pearson correlations between sensory processing (SP) Differences, repetitive motor behaviors (RMB), insistence on sameness behaviors (ISB), Alexithymia, Uncertainty, Anxious Arousal, Social Anxiety, Age, and Gender.

	SP Differences	RMB	ISB	Alexithymia	Uncertainty	Anxious Arousal	Social Anxiety	Age	Woman	Man	Qualification
SP Differences	—										
*N*	419										
RMB	0.656**	—									
*N*	415	420									
ISB	0.650**	0.605**	—								
*N*	416	417	420								
Alexithymia	0.359**	0.350**	0.277**	—							
*N*	416	416	415	418							
Uncertainty	0.403**	0.359**	0.521**	0.371**	—						
*N*	416	416	417	415	422						
Anxious Arousal	0.499**	0.458**	0.465**	0.396**	0.531**	—					
*N*	414	415	414	414	419	419					
Social Anxiety	0.253**	0.236**	0.325**	0.350**	0.485**	0.445**	—				
*N*	415	415	415	414	419	417	421				
Age	−0.031	−0.142**	0.07	−0.031	0.01	−0.111*	−0.116*	—			
*N*	419	420	420	418	422	419	421	426			
Woman	0.157**	0.05	0.027	0.048	0.117*	0.187**	0.123*	−0.091	—		
*N*	419	420	420	418	422	419	421	426	426		
Man	−0.188**	−0.071	−0.037	−0.055	−0.122*	−0.202**	−0.132**	0.123*	−0.949**	—	
*N*	419	420	420	418	422	419	421	426	426	426	
Qualification	0.040	0.028	−0.007	−0.064	0.002	0.047	−0.040	0.011	−0.019	0.198**	—
*N*	419	420	420	418	422	419	421	426	426	426	426

* Correlation is significant at the 0.05 level.

** Correlation is significant at the 0.01 level.

**FIGURE 2:**
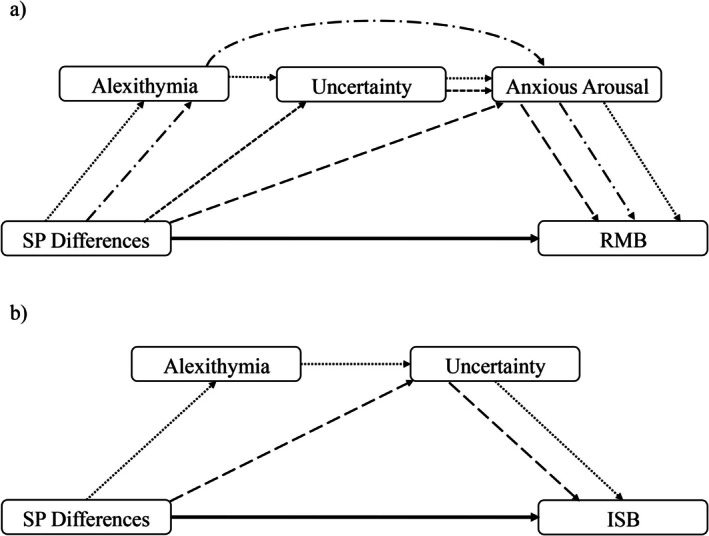
Significant direct and indirect pathways identified through mediation analyses for the relationship between sensory processing differences and repetitive motor behaviors and insistence on sameness behaviors, respectively.

All mediation outputs are displayed in Table 4. Significant pathways are presented in Figure 2. There were significant direct effects from SP differences on both RMB and ISB. For RMB, there was an indirect effect of Anxious Arousal alone, and there were a further three serial mediating relationships, all involving Anxious Arousal: Alexithymia ➔ Anxious Arousal; Uncertainty ➔ Anxious Arousal; and Alexithymia ➔ Uncertainty ➔ Anxious Arousal. All of the indirect effects involving social anxiety were nonsignificant. This model accounted for 48% of the variance in RMB scores. For ISB, Uncertainty was a single mediator, and there was a serial indirect effect through Alexithymia ➔ Uncertainty. None of the indirect effects involving Anxious Arousal or Social Anxiety were significant. This model accounted for 52% of the variance in ISB scores.

**TABLE 4:** Total, direct, and indirect effects of Alexithymia, Uncertainty, Anxious Arousal, and Social Anxiety as mediators between sensory processing (SP) Differences and repetitive motor behaviors (RMB) and insistence on sameness behaviors (ISB).

Outcome	Effect		*B*	SE	LLCI	ULCI
RMB (*N* = 409)	Total effect		0.1468*	0.0077	0.1316	0.1621
	Direct		0.1247*	0.0095	0.1060	0.1433
	Indirect effects	Total	0.0999*	0.0237	0.0540	0.1483
	*Through*	Alexithymia	0.0268	0.0154	−0.0019	0.0585
		Uncertainty	0.0160	0.0140	−0.0107	0.0447
		Anxious Arousal	0.0344*	0.0155	0.0061	0.0660
		Social Anxiety	0.0016	0.0039	−0.0057	0.0107
		Alexithymia ➔ Uncertainty	0.0051	0.0045	−0.0035	0.0146
		Alexithymia ➔ Anxious Arousal	0.0064*	0.0038	0.0008	0.0155
		Alexithymia ➔ Social Anxiety	−0.0015	0.0032	−0.0087	0.0041
		Uncertainty ➔ Anxious Arousal	0.0128*	0.0061	0.0023	0.0260
		Uncertainty ➔ Social Anxiety	−0.0023	0.0045	−0.0121	0.0061
		Anxious Arousal ➔ Social Anxiety	−0.0016	0.0032	−0.0085	0.0044
		Alexithymia ➔ Uncertainty ➔ Anxious Arousal	0.0041*	0.0022	0.0006	0.0092
		Alexithymia ➔ Uncertainty ➔ Social Anxiety	−0.0007	0.0015	−0.0039	0.0020
		Alexithymia ➔ Anxious Arousal ➔ Social Anxiety	−0.0003	0.0006	−0.0017	0.0008
		Uncertainty ➔ Anxious Arousal ➔ Social Anxiety	−0.0006	0.0012	−0.0033	0.0016
		Alexithymia ➔ Uncertainty ➔ Anxious Arousal ➔ Social Anxiety	−0.0002	0.0004	−0.0011	0.0005
ISB (*N* = 407)	Total effect		0.2108*	0.0119	0.1873	0.2342
	Direct		0.1693*	0.0145	0.1407	0.1979
	Indirect effects	Total	0.1316*	0.0288	0.0757	0.1881
	*Through*	Alexithymia	−0.0206	0.0163	−0.0527	0.0118
		Uncertainty	0.0796*	0.0184	0.0457	0.1172
		Anxious Arousal	0.0194	0.0151	−0.0094	0.0509
		Social Anxiety	−0.0035	0.0042	−0.0131	0.0036
		Alexithymia ➔ Uncertainty	0.0256*	0.0079	0.0116	0.0425
		Alexithymia ➔ Anxious Arousal	0.0034	0.0031	−0.0015	0.0108
		Alexithymia ➔ Social Anxiety	0.0039	0.0031	−0.0015	0.0109
		Uncertainty ➔ Anxious Arousal	0.0070	0.0058	−0.0033	0.0195
		Uncertainty ➔ Social Anxiety	0.0059	0.0044	−0.0021	0.0155
		Anxious Arousal ➔ Social Anxiety	0.0040	0.0029	−0.0016	0.0103
		Alexithymia ➔ Uncertainty ➔ Anxious Arousal	0.0023	0.0019	−0.0011	0.0065
		Alexithymia ➔ Uncertainty ➔ Social Anxiety	0.0019	0.0016	−0.0006	0.0056
		Alexithymia ➔ Anxious Arousal ➔ Social Anxiety	0.0007	0.0006	−0.0003	0.0020
		Uncertainty ➔ Anxious Arousal ➔ Social Anxiety	0.0014	0.0011	−0.0006	0.0038
		Alexithymia ➔ Uncertainty ➔ Anxious Arousal ➔ Social Anxiety	0.0005	0.0004	−0.0002	0.0014

*Note:* Indirect effects used bootstrapped SE, LLCI, and ULCI.

Abbreviations: LLCI, lower level confidence interval; SE, standard error; UCLI, upper level confidence interval.

* Effect is significant at the 0.05 level.

## 4 Discussion

This is the first study to consider IU, anxious arousal, social anxiety, and alexithymia when investigating the relationship between SP differences and RRB in autistic adults. All variables positively correlated with each other. Mediation analyses showed significant direct effects between SP differences and both RMB and ISB, respectively, supporting our prediction. Single and serial mediators of RMB were Anxious Arousal alone, and Alexithymia ➔ Anxious Arousal, Uncertainty ➔ Anxious Arousal, and Alexithymia ➔ Uncertainty ➔ Anxious Arousal. For ISB, these were Uncertainty alone, and Alexithymia ➔ Uncertainty. These findings demonstrate the importance of understanding an autistic individual's experience of alexithymia, IU, and anxious arousal, when supporting SP differences and RRB.

The direct effect between SP differences and RRB is well documented with children (Chen et al. 2009; Glod et al. 2019; Wigham et al. 2015), and more recently demonstrated in adults (Moore et al. 2021). This supports the notion that a function of RRB may be to act as a means of maintaining homeostasis in response to SP differences (Zentall and Zentall 1983). Recent evidence from autistic adults supports this, with reports of RRB acting, at times, as a self‐regulatory response to overstimulation (Charlton et al. 2021; Collis et al. 2022; Kapp et al. 2019; Steward 2015).

Anxious Arousal was a single mediator of the relationship between SP differences and RMB. This builds on a limited evidence base surrounding the links between SP and anxious arousal (Krusemark and Li 2012; Miller et al. 1999; Top et al. 2019). Alexithymia and Uncertainty played a role as serial mediators, in conjunction with Anxious Arousal (Alexithymia ➔ Anxious Arousal; Uncertainty ➔ Anxious Arousal; Alexithymia ➔ Uncertainty ➔ Anxious Arousal). These findings support the literature base broadly, indicating the relationship between SP, alexithymia, IU, and RMB (Liss et al. 2008; Maisel et al. 2016; Moore et al. 2021; Pickard et al. 2020). Moreover, while the majority of research to date is with non‐autistic populations, these results support the findings of Larkin et al. (2022), who showed that alexithymia and IU predict general somatic symptoms in autistic adults.

Turning to the relationship between SP differences and ISB, Uncertainty acted as a single mediator of this relationship, as well as a serial mediator, through Alexithymia ➔ Uncertainty. This corroborates the role of IU, previously identified in autistic children and adults (Glod et al. 2019; Hwang et al. 2020; Wigham et al. 2015), and the associations of alexithymia with these variables (Liss et al. 2008; Maisel et al. 2016; Pickard et al. 2020). The findings also directly replicate the indirect pathways identified by Moore et al. (2021), when measuring IU using an autism‐specific measure of anxiety.

While we did find significant correlations between Social Anxiety and all other variables, social anxiety played no mediating role in the relationship between SP differences and RMB or ISB. This is not wholly unexpected, given the mixed findings in the literature with regard to both SP (Black et al. 2017, 2023; Pickard et al. 2020) and RRB (Ben‐Itzchak et al. 2020; Schiltz and Magnus 2021). Several possibilities could explain these findings. SP differences may not be driving the relationship between social anxiety and IU, anxious arousal, and RRB. IU and anxious arousal have previously been shown to predict the avoidance component of social anxiety (Shapiro et al. 2020), while Spain et al. (2020) reported stimming to ease anxiety, hypervigilance to be able to manage impressions, as well as avoidance and planning as coping strategies for social anxiety. Alternatively, the effect may not be observed due to suppression of RRB, in response to perceived negative evaluations from others (Collis et al. 2022). Finally, social communication differences may be a greater predictor of social anxiety than SP differences in this population (Maddox and White 2015). Further research is needed to understand the role of these variables in relation to social anxiety.

Our findings are consistent with the possibility that there are distinct pathways leading from SP to RMB and ISB, respectively, in autistic adults, and that different RRB may serve to alleviate different anxiety symptoms in response to the sensory environment. RRB are directly related to SP differences, but it also appears that RMB acts as a means of managing anxious arousal, and ISB acts as a means of managing IU. Importantly, alexithymia and IU (individually and together) are only associated with the SP–RMB relationship through their relationship with anxious arousal, rather than directly through their relationship to SP differences and RMB. Similarly, alexithymia is only associated with ISB through its relationship with IU. Thus, these variables may only play a role through the generation of anxious arousal (for RMB) or IU (for ISB).

Considering the earlier work of Moore et al. (2021) using the HADS, these results broadly replicate the findings. Both studies demonstrated the same direct effects, as well as indirect effects on the SP–ISB relationship. Where they differ is in the indirect effects through SP–RMB. Our work confirms the importance of alexithymia and IU only through their relationship with anxiety, but identifies the specific component of anxiety as anxious arousal, clarifying these relationships beyond the ability of Moore et al.'s work using the HADS. Thus, the current work extends the findings of Moore et al. (2021), by delineating components of the anxiety experience that are important to autistic people, to create a greater understanding of their role in the relationship between SP and RRB.

What could this mean for support and intervention for autistic individuals? RRB may play an important role in managing the sensory environment and associated anxiety response to the environment for some people, and clinicians should consider this during assessment, formulation, and intervention for autistic people experiencing anxiety. Furthermore, autistic adults report perceived negative evaluation and lack of acceptance from others, which may lead to suppression of RRB (Charlton et al. 2021; Collis et al. 2022; Kapp et al. 2019; McCormack et al. 2022), and consequent increases in distress (Charlton et al. 2021; Collis et al. 2022). Support offered to autistic adults should consider this; for example, Kapp et al. (2019) found that acceptance could be gained with understanding, while McCormack et al. (2022) described how support from others can promote self‐acceptance, choice, and authentic expression of RRB. This suggests that an important route to supporting autistic individuals is through the promotion of societal awareness and knowledge of autistic characteristics, such as RRB. One way of doing this may be through increased awareness and training of the role of SP and RRB for people working with autistic individuals. This could include consideration of how relevant reasonable adjustments (e.g., change to environment; Brice, Rodgers, et al. 2021) could be implemented and how clinicians could be trained to appropriately adapt assessment, formulation, and intervention within psychological therapies for anxiety experienced by autistic people (Rodgers et al. 2023).

Given the potential importance of RRB for self‐regulation or as a coping strategy, the goal is not to remove RRB unnecessarily (indeed, autistic individuals report RRB as pleasurable as well as a coping strategy; Collis et al. 2022). However, increased RRB from that which is typical for that individual may indicate that they are struggling in other ways. Modifying the sensory environment, developing coping strategies, and interventions to reduce IU may all help the autistic person to manage their environment and response, and give more options for choice about whether they want (rather than need) to use RRB. In terms of the sensory environment, Pfeiffer and Kinnealey (2003) found that treatment for sensory defensiveness reduced this and associated anxiety in adults, while Unwin et al. (2021) reported that changes to sensory environments reduced RMB in autistic children. Both emphasized the importance of control and choice in the effectiveness of their interventions. RRB interventions have been developed for use with children, with preliminary evidence of efficacy, but these focus primarily on redirecting behaviors that impact engagement and functioning (Boyd et al. 2011), and developing parent understanding of RRB to differentiate between those that are potentially harmful and require intervention, from those that are neutral, helpful, or pleasurable (Grahame et al. 2015, 2021). Other strategies aimed at understanding emotions, self‐regulation, arousal, and IU have been developed to reduce anxiety (Parr et al. 2020; Spain et al. 2015; Wood et al. 2020), but only one small single‐case experimental design has included RRB as an outcome (and shown improvement with IU intervention), as far as the authors are aware (Rodgers et al. 2018). There is a clear need for further research to verify our findings in a therapeutic context.

A strength of this sample was a well‐balanced gender split (52% women, 45% men, and 3% self‐describing), and a wide range of SRS‐2 scores of autistic characteristics. This suggests generalization across the autistic spectrum and to both autistic men and women. While the proportion of women may not be representative of the autistic population (Loomes et al. 2017), this does fit with response rates to online surveys among autistic students (Ames et al. 2016; Anderson et al. 2018; Gelbar et al. 2014). However, we cannot generalize beyond our sample demographics, and our sample is predisposed toward those with anxiety, and potentially those with an interest in mental health (Rubenstein and Furnier 2021). While we did not collect data on IQ, educational attainment acted as a proxy and showed no significant relationship to key mediation variables. The internal validity of our results is likely robust, as responders and non‐responders from ASC‐UK did not differ on key characteristics such as autistic traits (Brice, Rodgers, et al. 2021).

It is important to acknowledge that this cross‐sectional, correlational work cannot prove causation. One example of this is in the relationship between SP and anxious arousal. While our evidence supports SP differences leading to anxious arousal, it is also plausible that anxious arousal might cause or highlight (and so worsen) SP differences, through hypervigilance to the surroundings (Green and Ben‐Sasson 2010). Sass et al. (2010) found an early attentional bias to and preferential processing of threatening stimuli in highly anxious arousal participants, compared to controls (*N* = 83 undergraduate students). Thus, feedback loops between SP and anxious arousal may exacerbate both, increasing the need for RMBs as a coping strategy. Longitudinal work could further understanding of the interplay between these experiences.

A measurement strength of this study was the use of an autism‐specific anxiety measure, to ensure that the items and constructs were appropriately tailored toward an autistic population (Rodgers et al. 2020). However, the SPQ (Kent 2014) cannot delineate hypo‐ and hyper‐reactivity when measuring SP differences. Although the stability of the factor structures is debatable, previous research using other SP measures (e.g., short sensory profile; McIntosh et al. 1999) has found differential relationships between sub‐factors of SP, mediating variables, and RMB and ISB (Boyd et al. 2010; Lidstone et al. 2014; Wigham et al. 2015). In the future, it would be useful to explore responsivity in these relationships.

In conclusion, RMB and ISB have distinct associations with SP differences and anxiety. RMB is associated with anxious arousal, alongside alexithymia and IU, and ISB is associated with IU, as well as alexithymia. These findings indicate that different coping strategies may be used in response to different anxiety experiences, and this should be explored when offering psychological support to autistic people, to ensure appropriately adapted interventions that meet the individual's needs. More broadly, support offered to autistic people, be it through psychological intervention, access to services, or in other areas (e.g., at interview), should ensure the availability of reasonable adjustments to accommodate SP differences, as well as differences in anxiety and RRB preferences associated with SP. Societal education is essential to support awareness and practical guidance about adjustments for autistic people. Future research directly investigating the therapeutic gains of strategies designed to support SP differences and anxiety may advance our understanding of support options further.

## Ethics Statement

A favorable ethical opinion was obtained from the NHS Health Research Authority (HRA) and Wales REC 5 (ref: 18/WA/0014).

## Consent

All participants provided informed consent.

## Conflicts of Interest

The authors declare no conflicts of interest.

## Data Availability

The data that support the findings of this study are available on request from Prof Jeremy Parr. The data are not publicly available due to privacy or ethical restrictions.
